# Ineffective Erythropoiesis in β-Thalassaemia: Key Steps and Therapeutic Options by Drugs

**DOI:** 10.3390/ijms22137229

**Published:** 2021-07-05

**Authors:** Filomena Longo, Andrea Piolatto, Giovanni Battista Ferrero, Antonio Piga

**Affiliations:** Department of Clinical and Biological Sciences, University of Torino, 10043 Torino, Italy; andrea.piolatto@unito.it (A.P.); giovannibattista.ferrero@unito.it (G.B.F.); antonio.piga@unito.it (A.P.)

**Keywords:** thalassaemia, ineffective erythropoiesis, erythroid precursors, iron dysregulation, foetal haemoglobin, new treatments, drug therapy

## Abstract

β-thalassaemia is a rare genetic condition caused by mutations in the β-globin gene that result in severe iron-loading anaemia, maintained by a detrimental state of ineffective erythropoiesis (IE). The role of multiple mechanisms involved in the pathophysiology of the disease has been recently unravelled. The unbalanced production of α-globin is a major source of oxidative stress and membrane damage in red blood cells (RBC). In addition, IE is tightly linked to iron metabolism dysregulation, and the relevance of new players of this pathway, i.e., hepcidin, erythroferrone, matriptase-2, among others, has emerged. Advances have been made in understanding the balance between proliferation and maturation of erythroid precursors and the role of specific factors in this process, such as members of the TGF-β superfamily, and their downstream effectors, or the transcription factor GATA1. The increasing understanding of IE allowed for the development of a broad set of potential therapeutic options beyond the current standard of care. Many candidates of disease-modifying drugs are currently under clinical investigation, targeting the regulation of iron metabolism, the production of foetal haemoglobin, the maturation process, or the energetic balance and membrane stability of RBC. Overall, they provide tools and evidence for multiple and synergistic approaches that are effectively moving clinical research in β-thalassaemia from bench to bedside.

## 1. Introduction

β-thalassaemia is an inherited monogenic disorder characterized by chronic anaemia caused by the reduced or absent production of functional haemoglobin (Hb). A broad spectrum of phenotype is observed in this condition, mainly defined by the degree of anaemia: transfusion-dependent β-thalassaemia (TDT) is characterized by a lifelong requirement for blood transfusions, while non-transfusion-dependent β-thalassaemia (NTDT) may require limited transfusions for a restricted period. Complications arise mainly due to the anaemia itself and to the iron overload typically observed in these patients.

**Figure 1:**
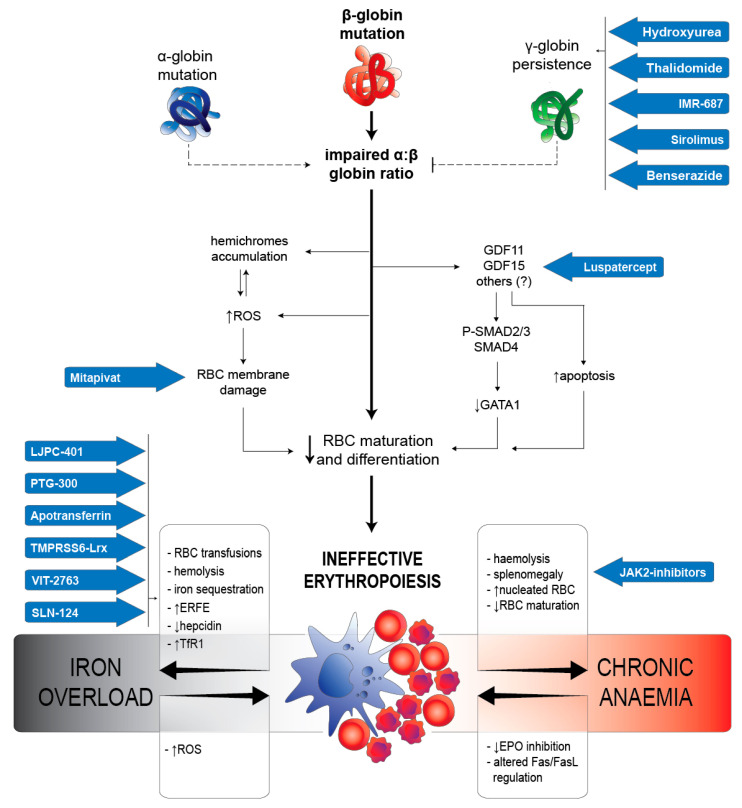
Schematic representation of mechanisms of ineffective erythropoiesis (IE) in β-thalassaemia. Blue arrows identify potential new treatments under investigation for this condition.

**Figure 2:**
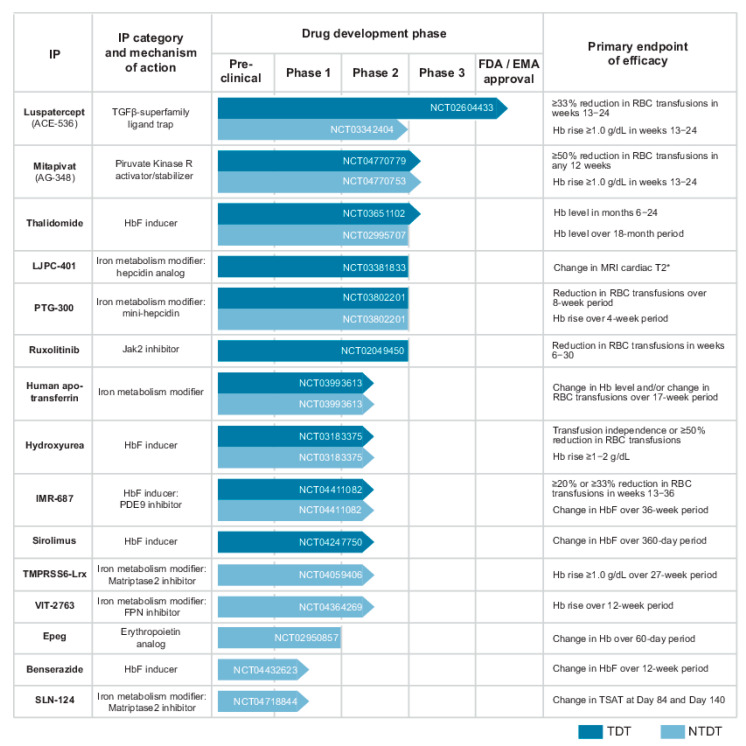
Development phase of investigational products (IP) that have been under clinical trial for the treatment of ineffective erythropoiesis in transfusion-dependent β-thalassaemia (TDT) or non-transfusion-dependent β-thalassaemia (NTDT), as registered on clinicaltrials.gov in the period 1 June 2016–31 May 2021. The identifier of the most recent trial is reported into the correspondent bar for each compound. Arrowed bar indicates an ongoing trial. FDA: U.S. Food and Drug Administration. EMA: European Medicines Agency.

In this narrative review, we describe new treatments for ineffective erythropoiesis (IE) in β-thalassaemia, focusing on the biological mechanisms that have been recently described and the pharmacological compounds that have been developed in this field of clinical research. In the first section, the main features of normal and altered erythropoiesis are briefly presented, alongside a description of the most relevant pathological and molecular mechanisms of IE. Particular emphasis was given to those processes that have a major role as possible targets of pharmacological modulation of this condition (Figure 1). In the second section, the approved or candidate new drugs for the treatment of IE in thalassaemia are presented, based on data available on https://clinicaltrials.gov/ (last accessed on 31 May 2021) regarding the last five years (1 June 2016–31 May 2021) (Figure 2).

## 2. Erythropoiesis

### 2.1. Steady-State and Stress Erythropoiesis in Physiological Conditions

Erythropoiesis is the physiological process that results in the production of red blood cells (RBC) at a constant rate under the regulation of a complex network of oxygen sensors and modulating cytokines that keep the Hb concentration at a remarkably stable level throughout the lifetime. The formation of mature RBC represents a highly regulated process that begins with the differentiation of multipotent haematopoietic progenitors in a single lineage [1]. After the commitment of the stem cell progenitors, erythroid precursors undergo an expansive phase of proliferation, followed by a stage of progressive maturation. Commonly described in a stepwise fashion, this is instead a continuous process characterized by progressive modifications in gene expression profiles toward the formation of mature erythrocytes [2,3,4]. These events are tuned by supporting and nursing macrophages, forming a functional structure named erythroblastic island that controls the crucial regulation of this complex balance [5]. The proliferative phase is highly dependent on erythropoietin (Epo), produced in response to hypoxic conditions by the activation of hypoxia-inducible factor (HIF-1α) and its downstream targets. Conversely, Epo has little influence on the maturation phase, which is controlled by a larger and only partially known group of paracrine and endocrine mediators involved in oxidative stress, inflammation, and apoptosis [6,7]. Moreover, in order to allow the synthesis of Hb, iron metabolism is tightly regulated throughout the whole process [8].

Under conditions of low oxygen delivery, i.e., in case of haemorrhage, haemolysis, high altitude, or acute anaemia, the rate of erythropoiesis may be expanded significantly to increase oxygen delivery to the tissues [9]. This physiological adaptation is defined as stress erythropoiesis. In such a situation, RBC production increases depending on Epo signalling, leading to the proliferation of stress erythroid progenitors, which are phenotypically and functionally different from the corresponding progenitors observed at steady-state [9]. The stress erythroid proliferation and differentiation require specific erythropoiesis macrophage-supporting activity that functions as a complement to the main pathway of Epo activation [10]. Conversely, in conditions of pathologically altered erythropoiesis, such as β-thalassaemia, the short life span and inefficient oxygen-carrying ability of the erythrocytes stimulate a detrimental state of stress erythropoiesis and favour the failure of effective RBC production [11].

### 2.2. IE in β-Thalassaemia

IE is the long-term result of a complex interplay of molecular mechanisms primarily involving the bone marrow and its articulate bidirectional crosstalk with the liver, spleen, and gut, eventually resulting in the production of pathological RBC. IE is the core driver of thalassaemia and the main cause of the majority of the clinical features of this pathology. In β-thalassaemia patients, bone marrow contains about six times more erythroid precursors than normal, and apoptotic cell death is about four times higher than in a healthy subject [12]. Apoptosis of the erythroid precursors, triggered by the relative excess of α-globin chains, leads to medullary and intravascular haemolysis [13]. In thalassaemia, the altered differentiation of erythroid progenitors seems to exacerbate IE, combined with increased proliferation and apoptosis [14], ultimately leading to anaemia, extramedullary haematopoiesis, splenomegaly, and systemic iron overload [15]. Advanced characterisation of the molecular bases of these complex processes is, therefore, essential for the development of effective disease-modifying therapies (Figure 1).

#### 2.2.1. RBC Alterations Caused by α:β Chains Impairment 

The genetic determinant of β-thalassaemia is the presence of a mutation in the β-globin gene that alters the physiological and most abundant form of Hb observed in adult life (HbA1), a tetramer resulting from two α- and two β-globin chains. The most common mutations involve the exon-coding sequence, but pathological sequence variants affecting the intronic or regulatory regions are not uncommon [16]. Overall, they result in a trunk or misfolded protein, categorized as β0 or β+, respectively. The former has a severely compromised activity and results in more severe phenotypes, while the latter shows the maintenance of a partial activity and is, therefore, associated with better clinical conditions [17,18]. In both cases, the number of β-globin chains available for coupling with the correspondent α-globins is reduced, resulting in an impaired ratio between α and β subunits and, consequently, in an increased number of unbounded α-chains. The α-subunits tend to bind haeme, forming highly insoluble aggregates, called haemichromes, that precipitate and bind or intercalate into the plasma membrane [19]. In addition, recent experiments performed using mild oxidizing conditions on healthy RBC suggested that haemichromes are formed as a result of progressive oxidation, even after the binding of globins to the cell membrane [20], and that they contribute to the promotion of oxidative stress through auto-oxidation and formation of reactive oxygen species (ROS) [21]. ROS located at the plasma membrane are indeed particularly toxic, since they are not readily accessible to the cytoplasmic antioxidant system and can easily oxidize membrane components, involving protein essential for RBC integrity and metabolism [21]. Alterations of the cell membranes are thought to induce RBC cell death, or erythroptosis, mainly due to changes in membrane permeability that cause less efficient production of ATP and decrease the overall lifespan of RBC, consequently resulting in increased haemolysis [19,22]. In this scenario, the synthesis of non-functional Hb with a high affinity for plasma membrane not only constitutes a considerable limit for effective oxygen-binding, but also represents a major source of oxidizing species production, further reducing RBC efficiency and lifespan and, therefore, contributing to untamed IE. In addition, pathologically high levels of free iron resulting from both increased haemolysis and altered iron metabolism (see dedicated section below) are converted into highly reactive hydroxyl and hydroperoxyl radicals [23]. The presence of an increasing amount of oxidative species alone is able to induce this reaction, amplifying the toxic effect on tissues, especially those more prone to iron-mediated oxidation [24]. Eventually, ROS originated from free iron contribute to long-term tissue alterations causing β-thalassaemia complications, including severe organ damage and even death [25].

In accordance with these mechanisms, genetic variants causing the persistence of foetal haemoglobin (HbF) during adult life are clearly associated with a milder phenotype and a decreased burden of thalassaemia [26,27]. In HbF, α-globins are indeed paired to γ-globins, decreasing haemichromes formation and, most relevantly, providing an effective alternative to HbA1 for oxygen transport.

#### 2.2.2. Iron Metabolism Dysregulation

Iron and erythropoiesis are linked in bidirectional crosstalk, playing a significant role in determining the typical features of IE in thalassaemia. On the one side, RBC progenitors that fail to mature increase the total iron pool of the body through direct and indirect mechanisms. On the other, the increased availability of iron favours the amplification of erythropoiesis itself. IE causes iron-overload through three complementary mechanisms. (1) The erythron is significantly expanded in beta-thalassaemia, and it consequently requires a larger amount of iron for its metabolism, up to 100-fold the physiological daily amount [28]. This request is met through the increased bone marrow production of erythroferrone (Erfe), which directly suppresses hepcidin [29,30]. Hepcidin, produced by hepatocytes, is the main regulator of iron homeostasis through the inhibition of ferroportin (Fpn), the only known iron exporter [31]. The inhibition of hepcidin mediated by Erfe allows iron absorption and recirculation, increasing its availability for physiological and pathological processes [29,32]. Besides, and independently of Erfe, hepcidin is regulated by matriptase-2, a transmembrane serine protease encoded by the gene TMPRSS6, whose inactivation by deletion or silencing improves IE in a mouse model of thalassaemia [33,34,35]. (2) A significant contribution to the total amount of circulating iron is due to haemolysis. The increased lysis of anisopoikilocytic RBC releases iron, both in haeme-bound and free form, contributing to tissue iron overload [36,37]. In agreement with this, recent evidence showed improved iron metabolism after increased RBC maturation resulting from pharmacologically recovered IE in β-thalassaemia patients [38]. (3) The expansion of the erythropoietic compartment depends on the expression of transferrin receptor 1 (TfR1) and transferrin receptor 2 (Tfr2) that modulate the iron supply to the erythron [39]. In thalassaemia patients and thalassaemia-trait carriers, serum TfR1 (sTfR1) is proportional to the amount of IE and is associated with lower hepcidin expression and increased iron overload [40]. In mice models of beta-thalassaemia, decreased expression of TfR1 has been associated with improved iron metabolism and IE [41]. TfR2, a constitutive component of the Epo receptor complex, is expressed during the differentiation of erythroid cells [42]. The knockout of TfR2 lowered Epo levels, and this reduction was associated with an increased proportion of mature precursors, a rise in Hb, and a decrease in reticulocytes and Erfe, marking improved erythropoiesis in a mouse model of thalassaemia [43].

On the other hand, iron overload itself affects the IE of thalassaemia. The virtually unlimited availability of iron represents a boost factor for erythropoiesis. In addition, toxic, free iron increases ROS production, diminishing RBC functionality and lifespan (see dedicated section above in the text). Multiple approaches have been developed to control IE through the fine tuning of iron metabolism, and different therapeutic options are emerging, based on the hypothesis that iron restriction could limit IE, thus improving clinical features of thalassaemia [44].

#### 2.2.3. Arrest of Maturation Mediated by TGF-β Superfamily

The transforming growth factor β (TGF-β) family recognises different molecules involved in extracellular signalling through the binding of transmembrane serine/threonine kinases type I or type II activin receptors [44,45]. The binding of their receptors activates the Smad pathway through phosphorylation and dimerization of Smad2–3, Smad 4, and other components. Activated Smad are able to translocate to the nucleus, where they act as transcriptional factors [46]. Among the TGF-β superfamily, the growth differentiation factor 11 (GDF11) and 15 (GDF15) are thought to have a prominent, yet not fully characterised, role in IE through the inhibition of RBC maturation, mediated by the activation of class II activin receptors A (ACVR2A) and B (ACVR2B). Today, they represent the best putative targets of the new ligand trap luspatercept (see the dedicated section in this review).

GDF11 role in thalassaemia was hypothesised because it is pathologically upregulated in splenic erythroblasts of thalassaemic mice (but not in mice under hypoxic or haemolytic anaemia conditions), and its drug-induced inactivation is associated with the improvement and promotion of terminal erythropoiesis [47]. GDF11 acts by activating the Smad2/3 pathway, eventually resulting in exacerbating clinical features associated with IE, such as lower Hb and increased ROS. These effects are mediated by a decrease in the nuclear localisation of GATA-binding protein 1 (GATA1), a transcription factor that plays a pivotal role in regulating erythropoiesis in mammals. In accordance with this observation, the inhibition of GDF11 by luspatercept partially restores GATA1 nuclear localisation and the expression of its downstream targets [48]. However, the surprising result that the absence of GDF11 alone is not able to improve the condition of IE questioned its role in erythroid maturation and in the biological effects of luspatercept treatment [49]. In addition to these observations, recent results showed that GDF11 is produced by early erythroid precursors, where it plays a role complementary to Erfe in hepcidin inhibition mediated by bone morphogenetic proteins (BMP) [50].

Elevated levels of GDF15 were observed in patients affected by thalassaemia syndromes and other disorders characterised by expanded erythropoiesis and correlated positively with markers of IE, such as Epo, soluble TfR1 (sTfR), and nucleated RBC levels, and with the clinical severity of the pathology itself [51,52,53]. GDF15 was recently shown to progressively increase during erythroid differentiation until its late phase and to negatively regulate erythroid cell growth, development, and proliferation. It also determines the downregulation of GATA1 and other transcription factors, while increasing apoptosis in vitro [54], thus stressing its prominent role in bone marrow expansion and IE. GDF15 was also indicated to contribute to the repression of hepcidin [51]; however, this function remains unclear and will need further confirmation.

#### 2.2.4. Regulating Proliferation/Maturation Balance by GATA-1

GATA1 is a transcription factor necessary for the correct maturation of the erythroid lineage. Acting through its pleiotropic targets, it is an essential regulator of the development of mature and functional RBC [55,56]. In a patient-derived cellular model of β-thalassaemia, the arrest of erythroblasts maturation was associated with reduced levels of GATA1 [57]. GATA1 is actively degraded through caspase-3-mediated cleavage [58], or preserved from this process by the chaperone HSP70 [59]. HSP70, however, tends to bind free α-chains, actively competing with other dedicated chaperones, such as α-haemoglobin-stabilizing protein (AHSP). In thalassaemia, the increased amount of unbalanced α-chains sequesters HSP70, leaving GATA1 accessible for caspase-3 cleavage. When this mechanism is experimentally inhibited, GATA1 expression is able to induce erythroid maturation, but not apoptosis [57]. The pathway of haeme-regulated inhibitor kinase (HRI) and eukaryotic translational initiation factors 2 (eIF2alpha) seems to play a relevant role in preventing denatured α-globin aggregates accumulation [60,61], indirectly favouring GATA1-mediated maturation. A distinct mechanism is mediated by the interaction between the apoptotic pathway of Fas ligand and its receptor, the activity of which promotes caspase-8 cleavage and consequent inactivation of GATA1 [44,58].

## 3. Therapeutic Options by Drugs

The recent advances in the understanding of the pathological mechanism of thalassaemia paved the way for development of a multitude of different therapeutic approaches that are currently under investigation worldwide. In the following section, we consider the investigational drug products that entered the clinical phase within the last five years, focusing on their current and future relevance for clinical practice (Figure 2). The actual standard of care of IE in thalassaemia, including blood transfusion and stem cell transplantation, as well as new therapeutic options based on gene therapies, are not considered in this review and are available elsewhere [62,63,64].

### 3.1. Luspatercept (ACE-536)

Luspatercept is the first disease-modifying drug for β-thalassaemia, currently approved by the US Food and Drug Administration (FDA) in 2019 and the European Medicines Agency (EMA) in 2020 for TDT patients. It is designed as a fusion protein that combines a modified extracellular domain of ACVR2B and the crystallisable fragment (Fc) region of human immunoglobulin IgG1 (modified ACVR2B-huIgG1) [47]. In this way, luspatercept acts as a ligand trap for extracellular signalling molecules that are part of the TGF-β superfamily, eventually resulting in the promotion of effective RBC maturation. After successful and encouraging evidence from previous clinical trials [65], a phase 3, double-blind, placebo-controlled study (NCT02604433) confirmed the efficacy of this drug in decreasing the transfusion burden of TDT patients [66]. Considering the safety profile, a higher risk of thromboembolic events was reported in these patients when treated with luspatercept, especially in the presence of other risk factors, such as splenectomy [66]. The recommended doses for thalassaemia patients are 1 or 1.25 mg/Kg, administered subcutaneously every 21 days. As confirmed by pharmacokinetic studies, this dosing scheme allows a stable plasma concentration to be reached over time, independently of factors such as age, sex, ethnicity, or renal or hepatic impairment [67]. Parallelly, a phase 2, double-blind, placebo-controlled trial of luspatercept in NTDT (NCT03342404) recently completed the blinded phase [68]. The positive results observed in this study in terms of efficacy and safety confirmed the relevance of luspatercept for these patients and could prompt its early approval for the treatment of NTDT.

### 3.2. Mitapivat (AG-348)

Mitapivat is an oral, small-molecule allosteric activator of RBC pyruvate kinase (PK-R) [69], a pivotal enzyme to regulate ATP production via glycolysis. In a phase 2 study on patients with pyruvate kinase deficiency, mitapivat administration resulted in a sustained Hb increase [70]. The hypothesis that increasing ATP synthesis via PK-R activation by mitapivat may also improve thalassaemic RBC fitness and survival was corroborated by interim results from an ongoing phase-2 study on α- and β-NTDT. Mitapivat showed a significant effect on Hb level and improved markers of haemolysis and IE in almost all patients [71], suggesting a promising role in the treatment of the late phase of IE. Among the reported adverse events, some could negatively affect the overall burden of the disease on thalassaemia patients, such as osteoporosis or hormonal alterations [70]. Particular caution will be necessary for addressing their relevance and causal relation to the study drug during later trials. Two phase-3 studies evaluating the efficacy and safety of mitapivat in patients with α- or β-TDT (NCT04770779) and NTDT (NCT04770753) have been recently started, but they are not yet recruiting patients at the time of this review.

### 3.3. Modifiers of Iron Metabolism

Improving iron dysregulation could represent an effective therapeutic strategy to control IE of thalassaemia. Several molecules were proved able to restrict iron availability to the erythron and improving RBC survival in preclinical studies and a few of them are currently under clinical trial. 

#### 3.3.1. Hepcidin Analogues

New molecules were developed with the aim of restricting the amount of iron delivered to the erythron by restoring physiological levels of hepcidin. Hepcidin analogues were able to ameliorate IE, anaemia, splenomegaly, and iron overload in NTDT model mice [72]. More recently, mini-hepcidin, in combination with chronic red blood cell transfusion, ameliorated IE, splenomegaly, and cardiac iron overload in a new model of TDT mice [73]. Human phase 1 studies showed a reduction in serum iron after following the administration of hepcidin [74,75]. LJPC-401 is a synthetic full-length hepcidin analogue of the mature form of the human hormone. It reached a phase 2 study, primarily aiming at evaluating the changes in iron levels in adult patients with TDT with myocardial iron overload (NCT03381833). However, an interim analysis involving half of the enrolled population suggested a lack of efficacy on the primary and secondary endpoints, leading to the early termination of the study. A slightly different approach was applied for the development of PTG-300, a hepcidin mimetic that shares only the N-terminal portion of the human peptide sequence [76]. PTG-300 was investigated in a phase II clinical trial involving both TDT and NTDT patients (NCT03802201), confirming the activity of PTG-300 in reducing TSAT and serum iron in a small number of subjects with TDT [77]. For both compounds, nonlimiting local injection site reactions appeared to be the chief adverse event. Altogether, although theoretically favourable and technically feasible, the direct administration of hepcidin, both in a complete or truncated form, did not show relevant benefits in the clinical setting until now. Different approaches to the modulation of iron metabolism target the upstream regulation of hepcidin and are now on a clinical trial.

#### 3.3.2. Apotransferrin

Transferrin (Tf) showed a critical role in the upregulation of hepcidin (HAMP) gene expression, degradation of Fpn in liver Küpffer cells, and correction of anaemia by Tf treatment in Hbb th1/th1 mice [78]. In addition, based on clinical evidence from a limited number of patients with congenital atransferrinemia, it appears that less than 0.5 mg/mL of Tf in plasma is sufficient to sustain adequate erythropoiesis [79]. Chronic apotransferrin administration in mouse models of TDT and NTDT resulted in normalisation of the anaemia. Furthermore, it normalised tissue iron content in the liver, kidney, and heart and attenuated early tissue changes in NTDT mice [80]. This effect was confirmed by the decreased expression of Erfe, increased liver and plasma hepcidin, and reduced intestinal Fpn in apotransferrin-treated thalassaemic mice [78]. An ongoing phase 2 trial (NCT03993613) will evaluate the effect of apotransferrin administration in a small sample of patients suffering from β-thalassaemia intermedia in restoring IE, measured by enhanced Hb levels or reduced transfusion dependency.

#### 3.3.3. Inhibitors of TMPRSS6

Among the strategies for controlling IE by modulating iron metabolism, the inhibition of matriptase 2 expression represents a novel approach in the clinical research scenario. After multiple proofs of concept of its efficacy on iron metabolism and erythroid differentiation in different models [33,34,81,82], TMPRSS6 inhibitors have recently entered clinical trials. TMPRSS6-Lrx firstly completed a double-blind, placebo-controlled, dose-escalation, phase 1 study on healthy volunteers (NCT03165864) and is currently on trial in an open-label, phase 2a study for patients affected by NTDT. Results from phase 1 confirmed a significant increase in hepcidin levels and an associated lowering of transferrin saturation (TSAT), without serious concerns for tolerability [83]. Phase 2 study will be crucial to assess not only the safety and efficacy of this compound, but also the clinical relevance of the overall approach in the correction of IE. Parallelly, SLN124 was developed based on a similar approach. After showing promising results in the preclinical phase [35], it has recently entered a first-in-human phase 1 trial on patients affected by NTDT (NCT04176653).

#### 3.3.4. Inhibition of Fpn by VIT-2763

VIT-2763 is a novel small molecule, orally administered, acting in the regulation of iron metabolism by inhibition of FPN. In vitro, VIT-2763 was effective in reducing iron efflux, showing an effect comparable to hepcidin in human macrophage and kidney-derived cell line. In vivo, VIT-2763 demonstrated a significant decrease in the levels of circulating iron and a synergistic effect with the iron chelator deferasirox [84]. The results of phase I study were consistent with preclinical data and showed dose linearity of VIT-2763 pharmacokinetic profile in healthy subjects [85]. A phase 2, randomised, double-blind, placebo-controlled parallel-group trial is currently ongoing (NCT04364269), evaluating the safety of the compound and its effectiveness in determining a rise in Hb over 12 weeks of treatment in patients affected by NTDT. If its efficacy and tolerability are confirmed, VIT-2763 alone or in combination with other drugs could play a significant role in the fine modulation of iron metabolism in IE.

### 3.4. Multiple Approaches to HbF Induction 

Since γ-globin can vicariate β-globin in the formation of a functional Hb tetramer, the induction of HbF synthesis in the post-natal life has been widely investigated as a potential strategy to treat thalassaemia. The persistence of HbF can indeed ameliorate the clinical severity of β-thalassaemia by reducing the degree of imbalance of α- to non-α-globin chains in those RBC precursors that are able to synthesise HbF in a sufficient amount, called F cells [86,87]. However, while numerous promising strategies and compounds have been studied in beta-thalassaemia, no universally effective agents have been found.

#### 3.4.1. Hydroxyurea

Hydroxyurea (also known as hydroxycarbamide) is the first drug approved for treating sickle cell anaemia. It acts through the inhibition of ribonucleoside diphosphate reductase, an enzyme essential for ribonucleotides synthesis, arresting cells at the G1 or S phase of the cell cycle [88]. Although the proper mechanism of action has not yet been clarified, this pharmacological activity eventually provides a selective advantage to the expansion of RBC containing higher levels of HbF [89]. Evidence suggested that hydroxyurea exerts a dose-dependent, bimodal effect on erythropoiesis by downregulating the expression of GATA1 and upregulating GATA2, and favours the Hb balance towards HbF by delaying RBC maturation and stimulating γ-globin expression [90]. Moreover, the main γ-globin gene repressor BCL11A is inhibited by hydroxyurea, and this promotes the reactivation of γ-globin and induction of HbF synthesis [91]. Several studies have shown that hydroxyurea inhibits IE in patients by decreasing the number of nucleated RBC or reducing soluble transferrin receptor levels [92]. Since this compound showed a reliable profile of safety and tolerability, it has been proposed for use in thalassaemia. Positive but highly variable effects of hydroxyurea were observed in TD and NTDT patients in terms of total Hb content and improvement of other RBC indices. A positive effect of hydroxyurea in reducing the risk of leg ulcers, pulmonary hypertension, and osteoporosis emerged from a study in a large cohort of NTDT patients [93]. Furthermore, several case reports showed efficacy of hydroxyurea in treating masses of extramedullary haematopoiesis [94]. However, robust and consistent data are still missing, and the usefulness of the compound in this pathology is still debated [95]. 

#### 3.4.2. Thalidomide

Thalidomide is commonly known for its immunomodulating and antiangiogenic activity [96]. Together with its derivate pomalidomide, it also exhibits a secondary, less characterised effect in increasing γ-globin expression, suggesting a possible role in the control of IE [97]. Besides isolated reports of safety and efficacy of this approach [98,99], the few studies conducted in the last years on patients affected by TDT and NTDT reported promising results in terms of Hb rise and transfusion independence [100,101]. However, these studies involved a very limited number of patients, and high variability was observed in baseline characteristics of the study populations, including extreme conditions such as baseline Hb ≤ 4.0 g/dL in 4 out of 25 patients. Most importantly, the follow-up period for adverse events and drug-related toxicity did not exceed 1 year, remaining insufficient to ensure the safety of this treatment. A recent study investigated the association of thalidomide and hydroxyurea in TDT patients and showed that almost half of them maintained Hb ≥ 9 g/dL without any transfusion for 6 months consecutively [102]. A high rate of adverse events was reported, including sedation and liver disease. Safety concerns were raised due to the risk of thrombosis associated with thalidomide [103]. For these reasons together, the use of thalidomide in beta-thalassaemia still needs robust confirmations. In the future, and in conditions of optimal management of the disease, thalidomide could represent an alternative to be carefully considered for exceptional cases in which standard or advanced-phase experimental treatments cannot be applied.

#### 3.4.3. IMR-687

IMR-687 is a highly selective and potent small-molecule inhibitor of PDE9. Although the precise mechanism needs to be fully clarified, the blockage of PDE9 acts to increase cGMP levels, which is associated with the reactivation of HbF. The effect of this compound was firstly proved in sickle-cell disease, resulting in a significant increase in HbF in phase 1 and early stage of phase 2 [104].

Considering the overall safety and efficacy profile of this compound already proved for a different indication, it has recently entered a phase 2b clinical trial in adult patients with TDT or NTDT (NCT04411082).

#### 3.4.4. Sirolimus

Sirolimus is an immunosuppressant agent, approved by the FDA for the prevention of rejection in transplant recipients for decades. In addition, the ability of this drug to induce γ-globin gene expression was observed and confirmed in erythroleukemia cell line and erythroid precursors cells in β-thalassaemia patients [105]. This evidence suggested a repurposing of sirolimus, which is now in a pilot, open-label phase 2 study (NCT04247750), aiming to evaluate the rise in HbF and its effect on IE and immune system in TDT patients.

#### 3.4.5. Benserazide

Benserazide is a small compound that has been recently added to the candidate drugs able to induce HbF expression. It was originally approved in its racemic form for the treatment of Parkinson’s disease to enhance plasma levels of L-dopa. The recently reported effect of chronic use of benserazide on HbF production suggested a possible repurposing of this drug for the treatment of haemoglobinopathies [106]. Preclinical studies confirmed a higher efficacy of the racemic form of this compound over each of its enantiomers and benserazide is currently undergoing a phase 1 study (NCT04432623) to evaluate the effects of administering 3 doses in NTDT patients [107].

### 3.5. EPEG

Polyethylene glycol-conjugated erythropoietin (EPEG) is a synthetic and highly stable analogue of Epo that has been suggested for the treatment of NTDT. The rationale for therapy with Epo is based on its relative insufficiency in regard to the degree of anaemia experienced in these patients. Hb increments with Epo in NTDT have been reported to be highly variable [108] and, in some cases, paradoxical iron supplementation was given to enhance the efficacy of Epo [109]. However, since Epo signalling acts on the expansion of the erythroid precursors and has little effect on the late stages of their maturation, this approach seems to challenge the actual model of IE in thalassaemia. The major concerns arise due to the risk of extramedullary erythropoiesis expansion, splenomegaly, and possible thrombotic events. After the completion of a phase 1 study (NCT02950857), no trials with Epo derivatives are currently ongoing.

## 4. Future Perspectives

The scientific and technical progress in the field brought us an unparalleled scenario of therapeutic opportunities, which are currently under clinical evaluation for the treatment of thalassaemia. The broader comprehension of the molecular foundation of IE that is continuously emerging has indeed allowed for the development of a large number of possible therapeutic approaches. From the clinical point of view, we suggest the following points for consideration in the next and rapidly evolving future of thalassaemia treatment. (1) The approval of disease-modifying drugs other than luspatercept will open the possibility of combining compounds acting on different yet interacting mechanisms of the disease and will, therefore, open novel opportunities, as already shown in mouse models [110,111]. Parallelly, it will also present the challenge of balancing multiple features of the pathology when deciding how to treat each patient. While, today, the total Hb levels and the TSAT are considered the most representative indices of IE, the introduction of new markers in the clinical practice, such as hepcidin, Erfe, or GDF15, among others, could facilitate an effective treatment tailoring. (2) Since multiple evidence are emerging about the role of the erythropoietic niche in the regulation of IE [112,113], innovative treatments targeting the thalassaemic bone marrow micro-environment could act synergistically with the compound here described to modulate IE of thalassaemia.
